# Is contact-line mobility a material parameter?

**DOI:** 10.1038/s41526-022-00190-y

**Published:** 2022-02-21

**Authors:** Jonathan M. Ludwicki, Vanessa R. Kern, Joshua McCraney, Joshua B. Bostwick, Susan Daniel, Paul H. Steen

**Affiliations:** 1 Robert Frederick Smith School of Chemical and Biomolecular Engineering, Cornell University, Ithaca, NY 14853 USA; 2 Sibley School of Mechanical and Aerospace Engineering Cornell University, Ithaca, NY 14853 USA; 3 Department of Mechanical Engineering, Clemson University, Clemson, SC 29631 USA

**Keywords:** Chemical engineering, Aerospace engineering, Mechanical engineering

## Abstract

Dynamic wetting phenomena are typically described by a constitutive law relating the dynamic contact angle *θ* to contact-line velocity *U*_*CL*_. The so-called Davis–Hocking model is noteworthy for its simplicity and relates *θ* to *U*_*CL*_ through a contact-line mobility parameter *M*, which has historically been used as a fitting parameter for the particular solid–liquid–gas system. The recent experimental discovery of Xia & Steen (2018) has led to the first direct measurement of *M* for inertial-capillary motions. This opens up exciting possibilities for anticipating rapid wetting and dewetting behaviors, as *M* is believed to be a material parameter that can be measured in one context and successfully applied in another. Here, we investigate the extent to which *M* is a material parameter through a combined experimental and numerical study of binary sessile drop coalescence. Experiments are performed using water droplets on multiple surfaces with varying wetting properties (static contact angle and hysteresis) and compared with numerical simulations that employ the Davis–Hocking condition with the mobility *M* a fixed parameter, as measured by the cyclically dynamic contact angle goniometer, i.e. no fitting parameter. Side-view coalescence dynamics and time traces of the projected swept areas are used as metrics to compare experiments with numerical simulation. Our results show that the Davis–Hocking model with measured mobility parameter captures the essential coalescence dynamics and outperforms the widely used Kistler dynamic contact angle model in many cases. These observations provide insights in that the mobility is indeed a material parameter.

## Introduction

Rapid wetting and dewetting are seen in a wide range of applications such as inkjet printing^1^ and 3D additive manufacturing^2^, the application of pesticides in the agricultural industry^3^, and the design of fuel tanks in microgravity environments. Such motions belong to the inertial-capillary spreading regime characterized by moderate Weber number $We={{{\mathcal{O}}}}(1)$ and small Ohnesorge number *Oh* ≪ 1. Inertial-capillary spreading can be described by the Davis–Hocking contact angle model with the contact-line mobility *M* a material parameter defined by the particular solid–liquid–gas system. In this paper, we provide a critical assessment of whether the experimentally measured mobility *M* is a material parameter through a combined experimental and numerical study of binary sessile drop coalescence without any fitting parameters.

**Fig. 1:**
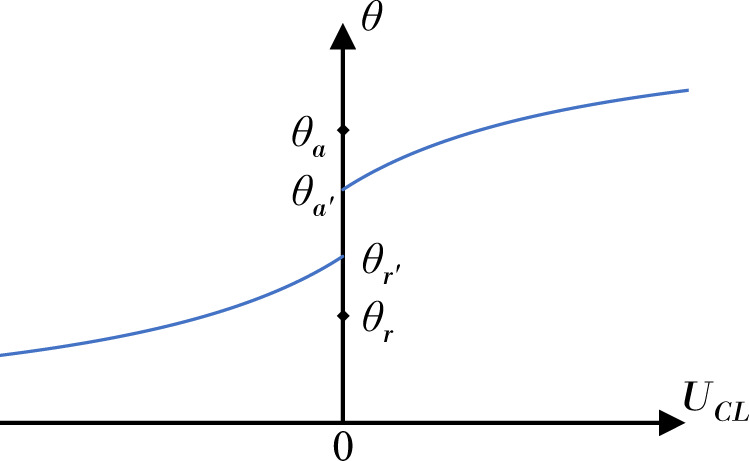
Traditional diagram for contact-line behavior. Dynamic contact angle *θ* dependence on contact-line speed *U*_*CL*_ with *θ*_a_ and *θ*_*r*_ the static advancing and receding contact angles, respectively.

The three-phase solid–liquid–gas contact-line sets the boundary between wet and unwet support^4^. For a spreading liquid, the contact-line advances across the solid with liquid displacing gas. Conversely, for a receding contact-line, the retreating liquid is displaced by gas. Contact-line motion, be it advancing or receding, has garnered significant research attention over the years due to its broad relevance in natural^5,6^, industrial^7,8^, and technological^9,10^ areas. One outcome of this research has been the development of a large number of proposed contact-line models^11,12^. Prominently used models include those based on classical hydrodynamic theory (Voinov^13^; Cox^14^), molecular kinetic theory (Blake & Haynes^15^), integrated hydrodynamic/molecular kinetic theory (Petrov & Petrov^16^), and empirical correlation (Kistler^17^). In most cases, the contact angle *θ* is related to the contact-line speed *U*_*CL*_, as shown in Fig. 1^18^. These models are required in any computational fluid dynamics simulation where two immiscible fluids, say water-air, interact with a solid surface^19–21^.

Of particular interest to this work is the so-called Davis–Hocking model and its potential for predictive capabilities in rapid wetting and dewetting regimes. First Davis^22^ and, later, Hocking^23^ viewed contact-line behavior as a single-valued function Δ*α* = *g*(*U*_*CL*_) with Δ*α* ≡ *θ* − θ^*^ and *g*(0) = 0. Linearization about the rest state Δ*α* = 0 yields the Davis–Hocking condition,

1 $$M{{\Delta }}\alpha ={U}_{CL},$$

with *M* the contact-line mobility parameter. Alternatively, *M*^−1^ can be viewed as a contact-line resistance. In previous literature works, *M* or *M*^−1^ has been referred to as a ‘phenomenological parameter’^24^, a ‘wetting parameter’^25^, the ‘Hocking coefficient’^26^, and the ‘mobility resistance parameter’^27,28^. Equation (1) can be modified to accommodate systems with contact angle hysteresis,

2 $$\theta ={\theta }_{a}+{U}_{CL}/M\,\,\,\,{{{\rm{for}}}}\,{U}_{CL} \,>\, 0,$$

3 $$\theta ={\theta }_{r}+{U}_{CL}/M\,\,\,\,{{{\rm{for}}}}\,{U}_{CL} \,<\, 0,$$

with *θ*_a_ and *θ*_*r*_ the advancing and receding contact angles, respectively. This approach is attractive for its simplicity, as well as for the ability to model contact-line behavior without the complications associated with possible contact angle multiplicity. However, determining the appropriate value of *M* for a particular solid–liquid–gas system is less straightforward, as typically *M* is varied in a simulation until a match to experiment is found, analogous to the contact-line friction factor tuning in Carlson et al.^29^. That is, *M* is determined via its application as a fitting parameter. Until recently, no direct technique for measuring *M* was available.

This has been addressed by Xia & Steen^30^, who have introduced a cyclically dynamic contact angle goniometer for measuring *M* in the inertial-capillary regime, defined by a competition between liquid inertia and surface tension, as characteristic in rapid wetting and dewetting phenomena. We note that over the past 50+ years, much work has been done on visco-capillary spreading and the associated contact-line singularity^31–35^. In contrast, relatively little work has been done on inertial-capillary spreading^36–38^ despite its relevance in applications, such as planar flow spin casting^39^ and immersion lithography^40^. In a recent review article, Snoeijer & Andreotti^41^ remark that, in terms of future issues related to understanding contact-line behavior, ‘new challenges for moving contact lines emerge from the influence of additional mechanisms, such as the inclusion of liquid inertia’.

To measure *M*, we emulate the experimental approach of Xia & Steen, using a mechanical shaker to drive the contact-line of a sessile drop via a plane-normal vibration of the drop’s support. The driving frequency corresponds to the [2, 0] resonance mode^42^. As the contact-line cyclically oscillates during the experiment (i.e. advancing and receding motions), measurements of Δ*α*, *U*_*CL*_, and contact-line displacement *η* are collected in time. By rescaling the data, a plot of *η*Δ*α* against *ηU*_*CL*_ reveals the inertial-capillary regime where Δ*α* is proportional to *U*_*CL*_ with slope *M*^−1^. An analogous conclusion has been reported by Fernández-Toledano et al.^43^ who found that, for a Lennard-Jones fluid, the uncompensated Young–Laplace force is linear in contact-line velocity via molecular dynamics simulations. It is further conjectured by Xia & Steen that, for a particular solid–liquid–gas system, *M* is a material-like parameter that can be first measured and then used to predict contact-line behaviors in other contexts, e.g. during drop impact, drop sliding, drop touching, or drop coalescence. However, the utility of *M* has yet to be confirmed.

In this paper, we evaluate whether *M* is a material parameter by first measuring M via the cyclically dynamic contact angle goniometer and then comparing binary sessile water drop coalescence experiments to simulations utilizing the measured *M*. Here four separate solid–water–air systems are used with widely varying wetting properties (static contact angle and hysteresis). Studies of coalescence were pursued due to both advancing and receding inertial-capillary contact-line motions during the merging process^44^. Furthermore, coalescence provides a challenging test of the mobility parameter for several reasons: (i) the occurrence of simultaneous advancing and receding motions, along with transitions between the two; (ii) the presence of contact-line motions that are not strictly inertial-capillary; (iii) the significant degree of contact-line motion and range of dynamic contact angle excursion during drop merging (Table 1). OpenFOAM is used to simulate the pairwise coalescence of identical sessile drops assuming the contact-line motion obeys either the (i) Davis–Hocking model with fixed *M* or (ii) Kistler model (Supplementary Material). We contrast experimental observations of the side-view coalescence dynamics and projected swept areas with numerical simulations and assess the validity of *M* as a material-like parameter.

**Table 1:** Comparison of experiments to test the mobility parameter for inertial-capillary contact-line motions.

Experiment	Simultaneous advancing and receding motions	Transitions between advancing and receding motions	Significant range of contact-line motion and *θ* excursion	Presence of non-inertial-capillary motions
Coalescence	✓	✓	✓	✓
Touching	✗	✗	✗	✗
Impact	✗	✓	✓	✓
Sliding	✓	✗	✓	✗
Vibration	✗	✓	✓	✓

## Methods

### Experiment

**Fig. 2:**
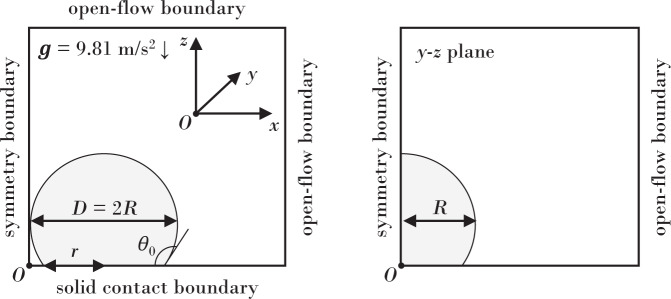
3D computational domain for simulating binary sessile drop coalescence. Symmetry planes as indicated.

Binary sessile water drop coalescence experiments were performed on four separate surfaces with wetting properties described in Table 2. We fabricated two hydrophobic surfaces (static contact angle *θ*_0_ ≈ 100^∘^, defined in Fig. 2) and two superhydrophobic surfaces (*θ*_0_ ≈ 150^∘^), which are further distinguished according to their contact-angle hysteresis, either low (Δ*θ* ≈ 5^∘^) or high (Δ*θ* ≈ 50^∘^). Here trimethylsilyl-terminated linear poly(dimethylsiloxane), or PDMS (DMS-T22, Gelest Inc.), was deposited on soda-lime glass (16004-422, VWR International) using the technique of Krumpfer & McCarthy^45^, while various Teflon samples (PTFENAT0, Ridout Plastics) were sanded using silicon carbide sandpaper of different grits (Starcke Abrasives) to produce the associated wetting properties. The mobility *M* values were measured via the method of Xia & Steen^46^. Specifically, unsanded Teflon underwent 57.7 Hz at 0.05 mm amplitude, Teflon 120 underwent 54.3 Hz frequency at 0.14 mm, amplitude, Teflon 240 underwent 46.9 Hz at 0.09 mm amplitude, and glass underwent 67 Hz at 3.4 mm. The experiments were performed using deionized water with liquid properties; density *ρ* = 998 kg/m^3^, surface tension *σ* = 0.072 J/m^2^, and viscosity *μ* = 0.998 mPa ⋅ s.

**Table 2:** Wetting properties of experimental surfaces defined by the static *θ*_0_, advancing *θ*_*a*_, and receding *θ*_*r*_ contact angles, contact angle hysteresis Δ*θ*, and mobility *M*.

Surface	*θ*_0_(^∘^)	*θ*_*a*_(^∘^)	*θ*_*r*_(∘)	Δ*θ* (^∘^)	*M* (m/rad ⋅ s)	*M* (Ca/rad)
PDMS on glass	109	109	106	3	0.24	0.003
Teflon (unsanded)	110	120	75	45	0.15	0.002
Sanded Teflon, 240 grit	151	152	148	4	0.31	0.004
Sanded Teflon, 120 grit	151	159	102	57	0.32	0.004

Coalescence was controlled by one of three methods. For PDMS on glass, a drop was moved via gentle blowing with air and made to coalesce with a second stationary drop. This method is analogous to the technique employed by Nilsson & Rothstein^47^. For unsanded Teflon and Teflon 120, coalescence was produced via surface through-hole growth of one drop until it touched a second pre-positioned drop. Finally, for Teflon 240, a trigger drop was pushed laterally into a second drop via a superhydrophobic wire, similar to the method of Wang et al.^48^. In all cases, care was taken to (1) impose a minimal impact velocity at the coalescence event and (2) ensure the drops were of approximately equal diameter *D* at the time of coalescence (within 10% of each other) with sizes in the range of 1.5 mm < *D* < 2.1 mm for each experiment. Top and side view video cameras (Redlake MotionPro HS-3 and Redlake MotionXtra HG-XL, respectively) simultaneously recorded the coalescence dynamics at a frame rate of at least 1000 frames per second.

### Numerical simulations

OpenFOAM computational fluid dynamics software (http://www.openfoam.com/) is employed to simulate the pairwise coalescence of identical sessile drops for the specific experiments described above. We briefly describe the procedure. Pre-processing is specified via blockMesh, a native OpenFOAM stencil tool, here used to generate a 2.6 mm × 2.6 mm × 2.6 mm cubic computational domain comprised of uniform cubic cells with edge length *ϵ* = 20 *μ*m, shown in Fig. 2. Increasing cell count by 25% yields <1% change in horizontal and vertical coalescence extensions; then the simulations are considered spacially converged with the chosen *ϵ*. Initial conditions are specified via the setFields utility, which specifies the initial cell value to a liquid or gas phase. Drop diameters *D*, static contact angles *θ*_0_ (c.f. Fig. 2), and parameters *θ*_*a*_, *θ*_*r*_, and *M*, are taken from experimentally measured values. Fluid properties are given in Table 3. Open-flow boundary conditions, i.e. fixed pressure and zero velocity gradient, are specified on all boundaries except the substrate. The gravity vector *g* is oriented in the negative *z*-axis direction with magnitude 9.81 m/s^2^, consistent with experimental conditions. Material advection, momentum, and continuity equations are solved for either the (1) Davis–Hocking or (2) Kistler dynamic contact angle model. The contact-angle model effectively prescribes the gradient of *α* according to the contact angle^49^. The numerical solution is computed via interFoam, a volume of fluid (VOF) solver, applicable to incompressible, laminar, and two-phase fluid flow. Here we note that the interFoam solver has been modified to remove artificial anti-diffusive surface fluxes, which have been previously shown to improve transient behaviors in capillary-dominated flows^50^. All post-processing is conducted in ParaView, a native software included with the OpenFOAM installation. The relevant non-dimensional numbers for the simulations are presented in Table 4 and are consistent with inertial-capillary spreading, *Re* ≫ 1, *Ca* ≪ 1, and *Oh* ≪ 1, for all cases.

**Table 3:** Liquid properties used in simulation for water (subscript *l*) and air (subscript *g*).

*ρ*_*l*_ (kg/m^3^)	*ρ*_*g*_ (kg/m^3^)	*μ*_*l*_ (mPa ⋅ s)	*μ*_*g*_ (mPa ⋅ s)	*σ*_*lg*_ (J/m^2^)
998.0	1.204	0.998	0.0181	0.072

**Table 4:** Non-dimensional characterization for simulations, as defined by the Reynolds *Re* = *ρ*_*l*_*U*_*CL*_*D*/*μ*_*l*_, capillary *Ca* = *μ*_*l*_*U*_*CL*_/*σ*_*lg*_, and Ohnesorge $Oh={\mu }_{l}/\sqrt{{\rho }_{l}{\sigma }_{lg}D}$. Here *U*_*CL*_ is the maximum magnitude of contact-line velocity in the *x*-axis for *y* = 0 and *z* = 0.

	Davis–Hocking			Kistler		
Surface	*Re*	*Ca*	*Oh*	*Re*	*Ca*	*Oh*
PDMS on glass	493	0.004	0.003	1491	0.012	0.003
Teflon (unsanded)	264	0.002	0.003	972	0.009	0.003
Sanded Teflon, 240 grit	851	0.006	0.003	1475	0.010	0.003
Sanded Teflon, 120 grit	615	0.004	0.003	1229	0.009	0.003

### Contact-line models

**Fig. 3:**
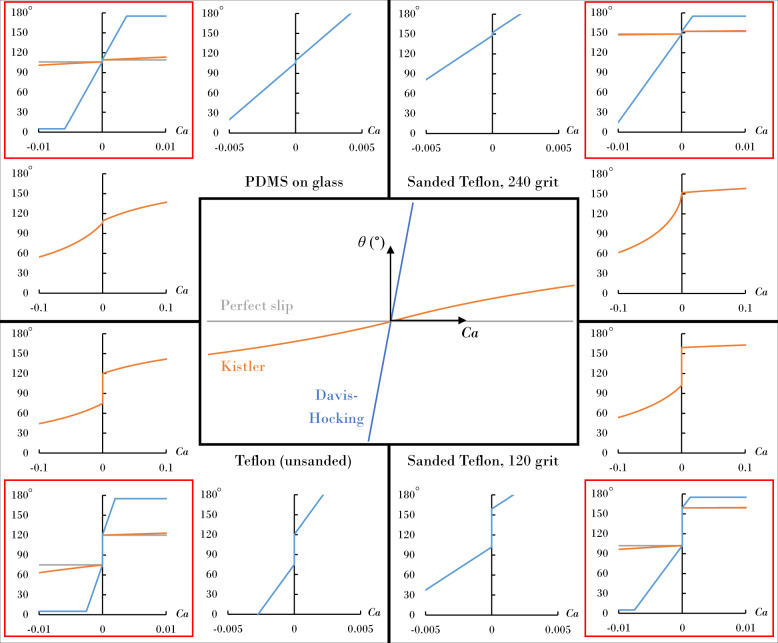
Family of contact-angle models plotting contact angle against capillary number *Ca*, contrasting the Davis–Hocking, Kistler, and perfect slip cases (core). The model responses shown on the perimeter incorporate contact-angle hysteresis for the surfaces used in this work. Those in the corners (red framed) indicate model behavior for the range of Ca explored in the current simulations. The other panels show model behavior for alternative *Ca* ranges.

The goal is here is to evaluate the efficacy of the Davis–Hocking condition, and the role of *M*, in reproducing the experimental results. We do this by performing simulations for both the Davis–Hocking model and Kistler model. Figure 3 contrasts the contact-line response, i.e. the contact angle *θ* against capillary number *Ca* ≡ *μ*_*l*_*U*_*CL*_/*σ*_*lg*_, for the family of contact angle models examined in this work. Here we note that the Kistler model exhibits contact-line motion close to perfect slip for the range of *Ca* observed during the experiment, which corresponds to a fixed contact angle *θ* = *θ*_*a*_ when advancing and *θ* = *θ*_*r*_ when receding. If we were able to access larger *Ca* in the experiment, then perhaps we could observe the nonlinearities in the Kistler model associated with large *Ca*.

The Davis–Hocking model, Equations (2) and (3), can yield non-physical dynamic contact angles for certain combinations of *U*_*CL*_, *M*, and *θ*_*a*_ (advancing case) or *θ*_*r*_ (receding case). That is, at large enough contact-line velocity, it is possible for the model to produce *θ* > 180^∘^ for an advancing contact-line or *θ* < 0^∘^ for a receding contact-line. Table 5 provides the range of *U*_*CL*_ values that maintain a physical dynamic contact angle where 0^∘^ ≤ *θ* ≤ 180^∘^ for the surfaces used in our experiments. To ensure the dynamic contact angle is physical 0^∘^ ≤ *θ* ≤ 180^∘^ in our simulations, we implement the following piecewise condition,

4 $${{{\rm{For}}}}\,{U}_{CL} \,>\, 0:\,\,\,\,\theta ={\theta }_{a}+{U}_{CL}/M\,\,\,\,{{{\rm{if}}}}\,\,\,\,\theta \,\le \,18{0}^{\circ },\,\,\,\,{{{\rm{otherwise}}}}\,\,\,\,\theta =17{5}^{\circ }$$

5 $${{{\rm{For}}}}\,{U}_{CL} \,<\, 0:\,\,\,\,\theta ={\theta }_{r}+{U}_{CL}/M\,\,\,\,{{{\rm{if}}}}\,\,\,\,\theta \ge {0}^{\circ },\,\,\,\,{{{\rm{otherwise}}}}\,\,\,\,\theta ={5}^{\circ }.$$

Here if the Davis–Hocking model calculates *θ* > 180^∘^ for *U*_*CL*_ > 0, then we set the dynamic contact angle to *θ* = 175^∘^, Equation (4). Similarly, when the model calculates *θ* < 0^∘^ for *U*_*CL*_ < 0, the dynamic contact angle is set to *θ* = 5^∘^, Equation (5). The current contact-line condition stipulates piecewise change at (5^∘^, 175^∘^) changeover, implied by (5). Increasing the piecewise condition to (1^∘^, 179^∘^) or decreasing to (10^∘^, 170^∘^) changes results <1%.

**Table 5:** Davis–Hocking model limits: range of *U*_*CL*_ values that produce a valid dynamic contact angle where 0^∘^ ≤ *θ* ≤ 180^∘^ for the parameter values presented in Table 2.

Surface	*U*_*CL*_ Range
PDMS on glass	−0.45 m/s < *U*_*CL*_ < 0.30 m/s
Teflon (unsanded)	−0.19 m/s < *U*_*CL*_ < 0.15 m/s
Sanded Teflon, 240 grit	−0.79 m/s < *U*_*CL*_ < 0.15 m/s
Sanded Teflon, 120 grit	−0.56 m/s < *U*_*CL*_ < 0.12 m/s

## Results

### Experiments and simulations

**Fig. 4:**
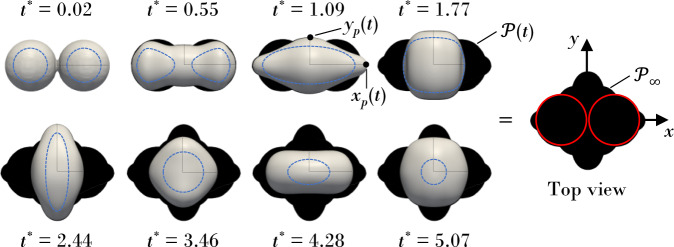
Extensional and swept areas during coalescence showing the evolution of the *x*-axis *x*_*p*_(*t*) and *y*-axis *y*_*p*_(*t*) projected extensions. The contact-line position (dashed blue line) is superimposed on the top view. Instantaneous cumulative projected swept areas ${{{\mathscr{P}}}}(t)$ are indicated by the black silhouettes, with the final cumulative projected swept area ${{{{\mathscr{P}}}}}_{\infty }$ shown on the rightmost image. The circles on ${{{{\mathscr{P}}}}}_{\infty }$ (red solid) represent the initial projected drop areas.

**Fig. 5:**
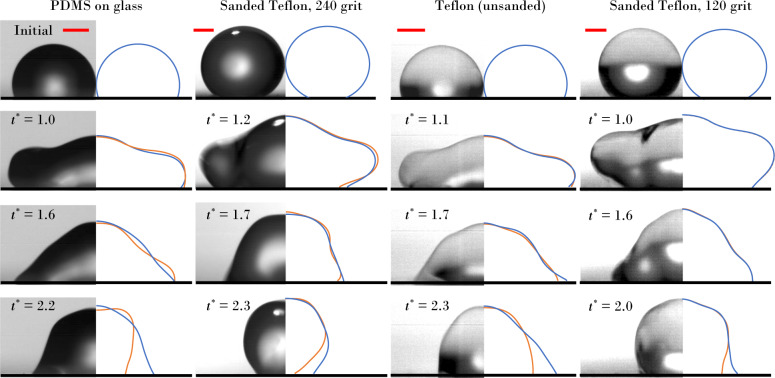
Comparison of side-view coalescence dynamics (*x*-*z* plane) contrasting experiments (left) with simulations (right). The Davis–Hocking model is shown in blue line type and the Kistler model in orange line type. Scale bar (red line) is 0.5 mm in all cases.

Here we assess the accuracy of the Davis–Hocking contact-line model against the Kistler model by comparing to experiment. Accuracy is assessed via three metrics: (i) time traces of the projected coalescence extensions in the *x*- and *y*-axes, *x*_*p*_(*t*) and *y*_*p*_(*t*) respectively (c.f. Fig. 4); (ii) the final cumulative swept droplet area ${{{{\mathscr{P}}}}}_{\infty }$ (c.f. Fig. 4); (iii) time evolution of side perspective coalescence event (c.f. Fig. 5). Parameters are nondimensionalized via

6 $${x}_{p} \sim 2R,\,\,\,{y}_{p} \sim 2R,\,\,\,t \sim \tau \equiv \sqrt{{\rho }_{l}{R}^{3}/{\sigma }_{lg}},\,\,\,{{{{\mathscr{P}}}}}_{\infty } \sim {{{{\mathscr{P}}}}}_{0}\equiv 2\pi {R}^{2}$$

where *R* is initial droplet radius. Hereafter, all dimensionless parameters are denoted with an asterisk (^*^).

Figure 5 contrasts the coalescence dynamics via side-view perspective (*x*–*z* plane) for the four surfaces. We focus on times t^*^ > 1, after which the capillary wave traverses the drop periphery, since this is when the contact-line begins to move and the motion is most rapid. For the low hysteresis surfaces (PDMS and Teflon 240), the Davis–Hocking model outperforms the Kistler model, which produces an overly mobile contact-line. In contrast, for the high hysteresis surfaces (Teflon and Teflon 120), the Kistler model outperforms the Davis–Hocking model, which excessively restricts the mobility of the receding contact-line, particularly at later times when the contact-line is receding. For inertial-capillary contact-line motions, the macroscopic dynamic contact angle is hypothesized to be unaffected by the macro geometry of the substrate due to the high inertia in the area of the contact-line. The success of each model on the various surface types likely comes down to the regime of contact-line behavior probed at any instant during the coalescence event. As seen in the data, the Kistler model is seemingly more appropriate for the visco-capillary regime because it was derived from Hoffman’s data at low Ca. In contrast, the Davis–Hocking mobility model derives from measurements of inertial-capillary contact-line motions and accurately describes the velocity dependence of the macroscopic dynamic contact angle in this regime. For inertial-capillary contact-line motions, the macroscopic dynamic contact angle is hypothesized to be unaffected by the macro geometry of the substrate due to the high inertia in the area of the contact-line.

**Fig. 6:**
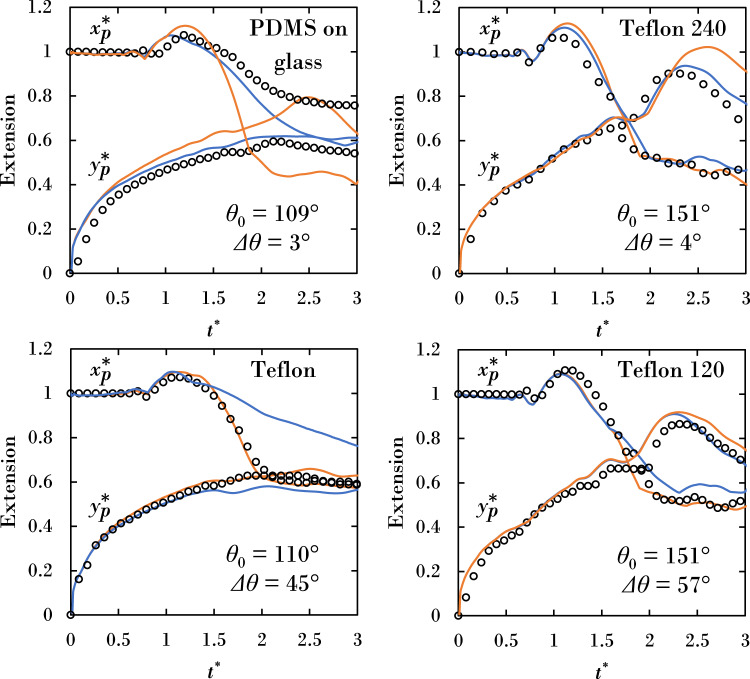
Time evolution of projected extensions contrasting experiments (open circles) with simulations (solid lines). The Davis–Hocking model is shown in blue line type and the Kistler model in orange line type.

Figure 6 plots the projected extensions ${x}_{p}^{* }$, ${y}_{p}^{* }$ for all surfaces. As expected from Fig. 5 the Davis–Hocking model outperforms the Kistler model for low hysteresis surfaces (PDMS and Teflon 240) for both ${x}_{p}^{* }$, ${y}_{p}^{* }$. Surprisingly, the Davis–Hocking model outperforms the Kistler model when predicting ${y}_{p}^{* }$ even on high hysteresis surfaces. Based solely on time traces of the ${x}_{p}^{* }(t)$, ${y}_{p}^{* }(t)$ extensions, the Davis–Hocking model outperforms the Kistler model for three out of the four surfaces (PDMS, Teflon 240, and Teflon 120). This is confirmed by the coalescence dynamics shown in Fig. 5, with the exception of the Teflon 120 surface, where the Davis–Hocking contact-line deviates from experiment at t^*^ = 2. Furthermore, the Davis–Hocking model better predicts the normalized cumulative projected swept areas (${{{{\mathscr{P}}}}}^{* }$), as shown in Table 6, for the same three surfaces. Overall, the comparison of the side-view dynamics gives the best indication of model performance versus experiment, with the coalescence metrics generally confirming those observations. We note numerically ±5% change in *M* yields < 2% change in change in ${x}_{p}^{* }$, ${y}_{p}^{* }$ extensions, and ±50% change in *M* yields <12%. It is also found changing the advancing/receding contact angles by ±2% from reported values in Table 2 yields <3% change in ${y}_{p}^{* },{x}_{p}^{* }$, and ±5% change yields <8%.

**Table 6:** Cumulative projected swept areas (normalized) for experiment and simulation.

Surface	${{{{\mathscr{P}}}}}^{* }$, Experiment	${{{{\mathscr{P}}}}}^{* }$, Davis–Hocking	${{{{\mathscr{P}}}}}^{* }$, Kistler
PDMS on glass	1.27	1.24	1.39
Teflon (unsanded)	1.31	1.26	1.30
Sanded Teflon, 240 grit	1.51	1.55	1.63
Sanded Teflon, 120 grit	1.50	1.51	1.53

## Discussion

In this paper, we study binary sessile water drop coalescence from an experimental and numerical perspective. The primary focus investigates whether an independently measured contact-line mobility parameter for inertial-capillary motions can be utilized in a Davis–Hocking type dynamic contact angle model to accurately capture experimental behaviors. That is, we determine if contact-line mobility is truly a material parameter or if it is system or geometry-dependent. Our results show that the Davis–Hocking model with measured mobility parameter adequately captures the coalescence dynamics and dynamic contact-line behavior for several surfaces with different wetting properties, outperforming the widely used Kistler dynamic contact angle model in many cases. This gives some indication that mobility is indeed a material-like parameter that can be measured in one context and used to predict in another.

It is noteworthy that the Davis–Hocking model, despite using *an M* value strictly appropriate for inertial-capillary contact-line motions, is able to reasonably capture the experimental dynamics and contact-line behavior at key time periods for many of the surfaces. As to why the Davis–Hocking model performs less optimally in predicting the receding contact-line motions on the higher contact angle hysteresis surfaces (Teflon and Teflon 120), it is possible that the *M* values for these receding motions are inappropriate. That is, on the higher hysteresis surfaces, it may be the case that the advancing and receding motions have different *M* values. Recall that the single *M* value used in the simulations was determined via a technique that averaged contributions from both advancing and receding contact-line behavior. Although not performed in this work, we remark that it is theoretically possible to extract different *M* values for the advancing *M*_a_ and receding *M*_*r*_ motions using the cyclically dynamic contact angle goniometer of Xia & Steen. Going forward, it would be valuable to understand if *M*_*a*_ ≠ *M*_*r*_ and how this would affect associated numerical simulations/predictions.

Regarding the utility of the Davis–Hocking model and the mobility *M*, future studies should investigate when the linearity between the dynamic contact angle and contact-line velocity breaks down in the inertial-capillary regime, as the cyclically dynamic contact angle goniometer presented by Xia & Steen is limited in the maximum velocity of the contact-line excursions that can be tested. For example, the Teflon surface exhibited contact-line velocities of −0.05 m/s < *U*_*CL*_ < 0.08 m/s during the experimental determination of the mobility parameter. Of course, it is possible for a contact-line to move with a velocity outside this range, thus putting into question the validity of the *M* value used for predicting dynamic contact angles. Furthermore, as the physical bounds of the dynamic contact angle (0^∘^ ≤ *θ* ≤ 180^∘^) are approached at higher contact-line velocities, the linearity of *η*Δ*α* versus *ηU*_*CL*_ could possibly breakdown.

Recently, related microgravity experiments of sessile drop coalescence proposed by our group were conducted aboard the International Space Station in November 2020. The advantage of these low-g experiments is magnification in both spatial and temporal scales to better resolve the contact-line dynamics during coalescence. More specifically, the capillary length $\ell =\sqrt{\sigma /\rho g}$ for water under terrestrial conditions is *ℓ* = 3 mm, whereas in microgravity it is *ℓ* ~ 1m can have size on the order of meters. The ISS experiments used drops of size 2*R* = 3 cm or 10x larger than those used in the experiments presented here. Drop mass is 1000x larger. This is particularly important in experiment to accurately resolve the contact-line dynamics, especially for hydrophobic surfaces. In addition, the inertial-capillary time scale ${t}_{c}=\sqrt{\rho {R}^{3}/\sigma }$ increases by 30x, which means the coalescence dynamics can be imaged using standard cameras, instead of high-speed cameras. These ISS experiments should provide further insights into the contact-line dynamics and the utility of the mobility parameter in predictive application for inertial-capillary motions, and will be the focus of a forthcoming paper.

Finally, the application of *M* to other systems that exhibit dynamic wetting would provide more confidence in its utility, specifically those that lie solely in the inertial-capillary regime (e.g. short-time dynamic wetting of a drop touching a surface). Sessile drop coalescence, with simultaneous advancing and receding motions (and transitions between the two), provides a demanding test of the mobility parameter especially given that some of the contact-line motions are outside the strictly inertial-capillary regime. Our results are sufficiently promising to suggest that the mobility *M* is a material property that can be measured independently and then used in numerical simulations for prediction. We hope that these results encourage other researchers to explore the use of the mobility parameter for modeling rapid wetting and dewetting behaviors via the Davis–Hocking model.

### Reporting summary

Further information on research design is available in the Nature Research Reporting Summary linked to this article.

## Supplementary information

Reporting Summary

Supplemental

## Data Availability

The corresponding author will make data made available upon reasonable request.
