# Supplementary material for: Is contact-line mobility a material parameter?
Source: NPJ Microgravity. 2022 Feb 21;8:6. doi: 10.1038/s41526-022-00190-y (PMC8861058; doi:10.1038/s41526-022-00190-y)
Supplement: Supplementary file 2 — Supplemental [file 41526_2022_190_MOESM2_ESM.pdf]

Supplemental Material: Kistler model

The Kistler model estimates the dynamic contact angle via an empirical function deduced from a systematic study of silicone oils displacing air in glass capillaries, as performed by Hoffman [1]. It has found wide application in the literature due to its accuracy in replicating experimental behaviors [2;3;4]. Dynamic contact angles are

$$\theta = f[f^{-1}(\theta_a) + \frac{\mu U_{CL}}{\sigma_{lg}}] \quad \text{for } U_{CL} > 0, \tag{1}$$

$$\theta = f[f^{-1}(\theta_r) + \frac{\mu U_{CL}}{\sigma_{lg}}] \quad \text{for } U_{CL} < 0, \tag{2}$$

with the empirical Hoffman function f defined as

$$f(x) = \cos^{-1}\left\{1 - 2\tanh\left[5.16\Big(\frac{x}{1 + 1.31x^{0.99}}\Big)^{0.706}\right]\right\}. \tag{3}$$

References

[1] Hoffman, R. L. A study of the advancing interface. I. Interface shape in liquid-gas systems. *J. Colloid Interface Sci.* **50**, 228–241 (1975).

[2] Roisman, I. V. *et al.* Drop impact onto a dry surface: Role of the dynamic contact angle. *Colloids Surfaces A* **322**, 183–191 (2008).

[3] Moghtadernejad, S., Tembely, M., Jadidi, M., Esmail, N. & Dolatabadi, A. Shear driven droplet shedding and coalescence on a superhydrophobic surface. *Phys. Fluids* **27**, 032106 (2015).

[4] Xu, J., Chen, Y. & Xie, J. Non-dimensional numerical study of droplet impacting on heterogeneous hydrophilicity/hydrophobicity surface. *Int. J. Heat Mass Transf.* **116**, 951–968 (2018).
